# Does point-of-care ultrasound examination by the general practitioner lead to inappropriate care? A follow-up study

**DOI:** 10.1080/02813432.2025.2487095

**Published:** 2025-04-10

**Authors:** Camilla Aakjær Andersen, John Brandt Brodersen, Jan Mainz, Janus Laust Thomsen, Ole Graumann, Thomas Løkkegaard, Martin Bach Jensen

**Affiliations:** ^a^ Center for General Practice at Aalborg University, Aalborg, Denmark; ^b^ Centre of General Practice, Department of Public Health, Faculty of Health Sciences, University of Copenhagen; ^c^ Research Unit for General Practice, Region Zealand, Denmark; ^d^ Research Unit for General Practice, Department of Community Medicine, Faculty of Health Sciences, UiT The Arctic University of Norway, Tromsø, Norway; ^e^ Danish Center for Health Services Research, Department of Clinical Medicine, Aalborg University, Aalborg, Denmark; ^f^ Psychiatry, Aalborg University Hospital, Aalborg, Denmark; ^g^ Department of Health Economics, University of Southern Denmark, Odense, Denmark; ^h^ Department of Radiology, Aarhus University Hospital, Denmark

**Keywords:** Ultrasonography, primary care, quality assurance, general practice, adverse effects

## Abstract

**Background:**

The use of point-of-care ultrasound (POCUS) in general practice increases, but little is known about potential unintended findings and harms to patients. Information regarding such unwanted effects may be obtained by evaluating the medical records of patients who have been scanned by their general practitioner.

**Objective:**

To identify and characterize re-consultations related to POCUS use in general practice, potential misdiagnosis, overdiagnosis, and incidental findings, and to compare potentially troublesome cases to GPs’ scanning competence and type of ultrasound device.

**Design and Setting:**

Professors in general practice with extensive experience in both research and quality assurance in general practice did a blinded review of prospectively collected routine electronic medical record data combined with cross-sectional data collected in relation to POCUS examinations.

**Subjects:**

Twenty general practitioners collected data on 564 patients examined with POCUS in primary care.

**Main Outcome Measures:**

International standards for the classification of adverse events and incidental findings were used. First, research assistants identified all re-consultations described in the medical records that were related to the primary health complaint at the index consultation. Second, these re-consultations were classified by the medical experts in terms of seriousness and relation to the POCUS examination performed at the index consultation. In addition, the experts identified possible misdiagnosis, possible overdiagnosis, and incidental findings. Finally, identified cases were discussed in terms of appropriateness and described narratively.

**Results:**

Medical records of 564 patients were reviewed. A low risk of possible misdiagnosis (5.3%), potential overdiagnosis (0.7%), and incidental findings (0.7%) were found. Eleven POCUS-related re-consultations were identified and described.

**Conclusion:**

POCUS scanning performed by general practitioners was generally safe, but it can result in unnecessary examinations and potential harm in a few cases. Certain areas, e.g. pelvic scans that included the ovaries, may especially be prone to misdiagnosis.

**Trial Registration Number:**

NCT03375333

## Background

Ultrasonography (US) is increasingly used as a bedside point-of-care examination (POCUS) [1]. POCUS may lead to earlier and more correct diagnoses [2,3]. However, there are numerous safety aspects to be aware of [4]. US is a user-dependent technology that entails the risk of misinterpretation, which can lead to adverse events. Furthermore, imaging may lead to spurious findings, misdiagnosis, overdiagnosis, incidental findings, and the diagnosis of clinically unimportant conditions [5,6]. These effects may harm patients and increase healthcare costs [7]. These problems may be even more pronounced in general practice compared to a hospital setting due to challenges regarding training and supervision, a patient population with low pre-test probability for somatic pathology [8], and a waiting time for follow-up on imaging results which may cause a delay in diagnosis by weeks/months compared to hours/days in an in-acute hospital setting.

Literature on the use of POCUS in general practice is sparse and there is a lack of high-quality studies. A literature review [9] showed that the primary focus in previous research has been on training and the diagnostic accuracy of POCUS examinations in cross-sectional designs performed by general practitioners (GPs). Less attention was given to the patient’s clinical course and prognosis succeeding the GPs’ POCUS examination or the number of adverse events following the use of POCUS in general practice. However, false positives and false negatives were described in 17% and 16% of the included papers, respectively, whereas incidental findings were described in 20%. Hence, POCUS use in general practice might cause harm to patients.

As the use of POCUS in general practice increases [10–12]. So does the need for knowledge of potential unintended findings and harms to patients when GPs perform POCUS. Information regarding such unwanted effects could be obtained by evaluating the medical records of patients who have been scanned by their GP [13].

Therefore, the aim of this six-month follow-up study was to identify and characterize re-consultations described in the medical records that were related to the initial POCUS examination as well as potential ­misdiagnosis, overdiagnosis, and incidental findings following the use of POCUS in general practice. In addition, we aimed to describe the relationship between re-consultations, potential misdiagnosis, overdiagnosis, and incidental findings and GPs’ scanning competence and type of ultrasound device used.

## Method

Data for this follow-up study originates from a prospective cohort study that took place in Danish general practice clinics in 2018 [14]. In this study, 20 GPs included all patients examined with POCUS for one month. At the index consultation in general practice (between January and August 2018), where the patients were examined with POCUS, GPs collected data related to the POCUS exam, and patients were asked to provide informed consent to the collection and evaluation of their medical records after six months.

## Study type

A review of prospectively collected routine electronic medical record data combined with cross-sectional data collected in relation to the initial POCUS examination.

## Eligibility criteria

Participating GPs were purposely selected to reach a maximum variation in general practice organisation, geography, equipment, and experience both regarding seniority and POCUS experience. All patients who were examined with POCUS were asked to participate in the study. Patients were excluded if they did not wish to participate or if they did not give informed consent or if their medical record was unavailable, e.g. if they had changed GP.

Patients could only be included once and only patients assigned to participating clinics’ lists could participate in the study.

## Setting

Denmark has a public healthcare system, where consultations are free for patients [15]. Patients are listed with a specific GP clinic for primary healthcare and these clinics act as gatekeepers for secondary healthcare. The GPs document actions and treatments in the electronic medical records, and as standard care and communication, they receive notice of all health events regarding a patient on the list, e.g. lab results or discharge notices. All data are automatically uploaded to the patient’s electronic medical record. Hence, the GPs’ medical records contain a comprehensive overview of a patient’s pathway in the healthcare system. Still, there is no standard for the documentation in the medical records and often GPs restrict their documentation to short free-text sentences.

At the time of this study, no guidelines or regulations was supporting the use of POCUS in general practice and only a small group of early adopters was using this technology [16].

## Data collection and procedure

The data collection at the index consultation has been previously reported [14]. For this study, we included the following information related to the participating GP: Individual scanning competence assessment performed by a US expert at baseline (measured as an objective structured assessment of ultrasound skills score between 9 and 45) and the GP’s type of ultrasound device (low-end, mid-range and high-end). We also included the following information related to the POCUS examination registered by the GP using an online questionnaire during the index consultation: Organs scanned (we did not use predefined scanning protocols; instead GPs were asked to register all organs included in their POCUS exam), change in patient management following POCUS (change in plan for referral and/or change in treatment) and classification of POCUS findings (certain finding/uncertain finding/incidental finding).

Six months after the index consultations, the GPs collected and pseudo-anonymised all data from the electronic medical records of the participating patients using unique patient-ID numbers. The data was transferred to the research team between December 2018 and September 2019, either printed or saved on a USB stick.

### Administrative data cleaning

Initially, the medical records were screened by the principal investigator (CAA) and a research assistant to ensure (1) informed consent from the patient, (2) total anonymization of the medical records, (3) correct medical records matching the patient-ID, and (4) a digitalization of the medical records.

### Sorting review

Two research assistants (a medical student and a master in medical science with industrial specialization) individually reviewed all medical records to identify (1) the date of the index consultation, (2) the note describing the POCUS performed at the index consultation, and (3) any re-consultations for the same complaint defined as descriptions of subsequent encounters in primary- or secondary healthcare related to or possibly related to the health complaint raised in the index consultation. Both reviewers had experience with reading medical records and doing research.

Medical records identified by one or both reviewers as describing re-consultation related to or possibly related to the primary health complaints were sent on for an expert review. Deselected medical records (i.e. those with no encounters for the same complaint) were screened by one expert reviewer (MBJ) to ensure that all relevant re-consultations were identified and included.

### Classification based on experts’ review

**Figure 1.**
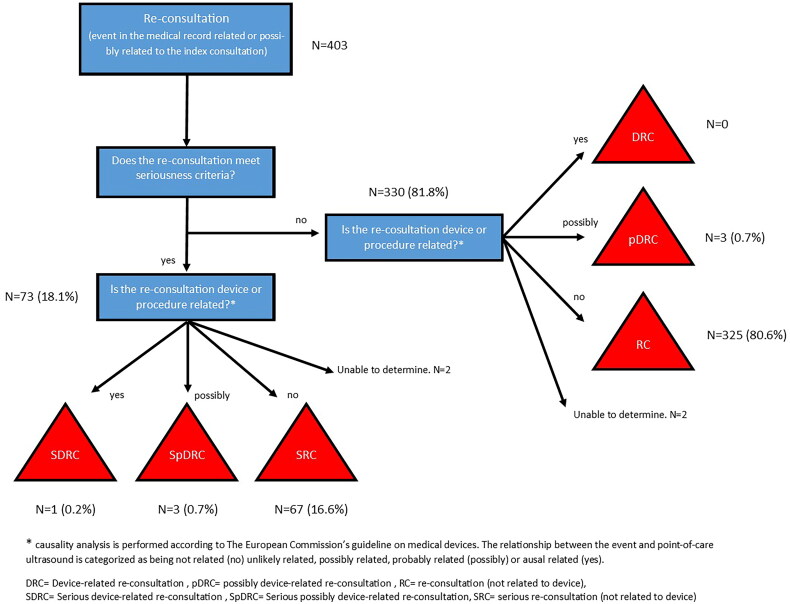
Classification of re-consultations.

The expert reviewers were GPs and professors in general practice with extensive experience in both research and quality assurance in general practice (MBJ, JB, NB, JLT). These expert reviewers reviewed and classified the identified medical records describing re-consultations for the same complaint. We adopted international standards for identifying and classifying adverse events to our classification of re-consultations (Box 1). Each re-consultation was classified in terms of seriousness [17] and causality (relation to the POCUS examination performed at the index consultation) [18] (Figure 1). In addition, the experts were asked to identify possible misdiagnosis, potential overdiagnosis, and incidental findings. The medical records were reviewed twice, and classifications were made independently by two experts. After that, the principal investigator (CAA) compared the two sets of classifications and invited the two experts to discuss inconsistencies. If agreement could not be reached, a third expert with extensive experience in working with quality assessment and improvement (JM) was involved.

### Consensus meeting

Following the identification and classification of re-consultations in the medical records, all experts met in a consensus meeting to discuss re-consultations and formulate a narrative description of (1) serious re-consultations related to the use of POCUS in general practice, (2) serious re-consultations possibly related to the use of POCUS in general practice, (3) non-serious re-consultations related to the use of POCUS in general practice, and (4) non-serious re-consultations possibly related to the use of POCUS in general practice. In addition, the experts classified the net benefit of incidental findings according to established standards [19] (Textbox 1).9

**Box 1.** Definitions and classifications.

**Table:** 

Classification of re-consultations for the same complaint described in medical records*	
Re-consultations for the same complaint	Serious re-consultations for the same complaint
Any re-consultation described in the medical record related or possibly related to the primary health complaint presented at the index consultation including both re-consultations in primary or secondary care for the same or similar complaint, follow-up consultations for the same complaint or test-results related to the primary complaint.	Re-consultations for the same complaint leading to (1) death, injury or permanent impairment to a body structure or a body function. (2) serious deterioration in health of the subject, that either resulted in: (a) a life-threatening illness or injury, (b) a permanent impairment of a body structure or a body function, (c) in-patient hospitalization or prolongation of existing hospitalization, (d) in medical or surgical intervention to prevent life threatening illness (3) led to fetal distress, fetal death or a congenital abnormality or birth defect.
POCUS related re-consultations**	Serious POCUS related re-consultations**
Re-consultations causally related to POCUS.	Serious re-consultations causally related to the use of POCUS.
Possibly POCUS related re-consultations**	Serious possibly POCUS related re-consultations**
Re-consultations unlikely related, possibly related, or probably related to the use of POCUS.	Re-consultations unlikely related, possibly related, or probably related to the use of POCUS.
Possible misdiagnosis	
Any inconsistencies drawn from the medical records between POCUS findings in general practice at the index consultation and repeated examinations in general practice or secondary care, where examinations can be compared. This includes both false-positives and false-negatives as well as any misclassification.	
Potential overdiagnosis***	
Any possible overdiagnosis made through over-detection or over-definition of disease. Over-detection refers to the identification of abnormalities that were never going to cause harm, abnormalities that do not progress, that progress too slowly to cause symptoms or harm during a person’s remaining lifetime, or that resolve spontaneously. False-positive results are not included in this definition.	
Incidental findings****	
Any incidental findings either registered by the participating GP or by the expert reviewers. Incidental findings were classified and described according to their net benefit as either having a *strong net benefit (*important incidental finding revealing a condition likely to be life-threatening or grave, that can be avoided or ameliorated), *possible net benefit* (incidental finding revealing a nonfatal condition that is likely to be grave or serious, but cannot be avoided or ameliorated) and *unlikely net benefit* (incidental finding revealing a condition that is not likely to be of serious health or reproductive importance e.g. detection of a US findings of doubtful clinical significance recorded by the GP at the consultation where POCUS was initially performed and sustained as of doubtful clinical significance at the evaluation after six months).	

* The international standard EN 540/ISO 14155 (https://www.iso.org/standard/45557.html).

** The European Commission’s guideline on medical devices(ref).

*** Brodersen J, Schwartz LM, Heneghan C, et al. Overdiagnosis: what it is and what it isn’t. BMJ Evidence-Based Medicine 2018;23:1-3.

**** Wolf SM, Lawrenz FP, Nelson CA, et al. Managing Incidental Findings in Human Subjects Research: Analysis and Recommendations J Law Med Ethics. 2008 ; 36(2): 219–211.

## Outcome measures

In this article, the number and frequencies of re-consultations for the same complaint are reported in relation to organs scanned and participating GP. The re-consultations are reported as either (1) Re-consultations related to the initial POCUS examinations (serious device-related re-consultations, serious possibly device-related re-consultations, Device-related re-consultations, and possible device-related re-consultations) or (2) Re-consultations not related to the initial POCUS examination (serious re-consultations and re-consultations) (Figure 1). Re-consultations for the same complaint related to the initial POCUS examinations are further described in relation to the GP’s change in patient management, certainty in findings, scanning skills, and type of POCUS device used. Finally, these events are elaborated narratively in terms of inappropriateness.

The number of medical records, including a description of possible misdiagnosis, potential overdiagnosis, and incidental findings are reported in relation the frequency of use and organ scanned. Incidental findings are further reported in relation to their net benefit.

## Data management

All data were handled according to the General Data Protection Regulation and national Danish laws—monitored by the Danish Data Protection Agency. A data processor agreement was made between the Research Unit for General Practice in Aalborg and each participating GP, between the Research Unit for General Practice in Aalborg and Aalborg University, and between Aalborg University and SurveyXact according to the Danish Data Protection Agency recommendations.

Printed copies of the electronic medical record were kept in a locked safe in a restricted access area. After the review process, the papers were converted to PDF files, which were safely stored at Aalborg University’s secure server. The patient key file connecting the patient ID with identifiable information and the signed consent forms were locked up at the GPs’ offices, and the research group did not have access to this information.

## Statistical methods

The identified re-consultations, cases of possible misdiagnosis, potential overdiagnosis, and incidental findings are described in absolute numbers and frequencies. The relationships between potentially troublesome cases (POCUS-related re-consultations, possible misdiagnosis, potential overdiagnosis, and incidental findings) and scanning competence are evaluated graphically, with each case related to the competence of the corresponding GP’ scanning competence at baseline (20–25, 26–30, 31–35, 36–40, 41–45) and the number of performed scans/included medical records in this study.

## Results

**Figure 2.**
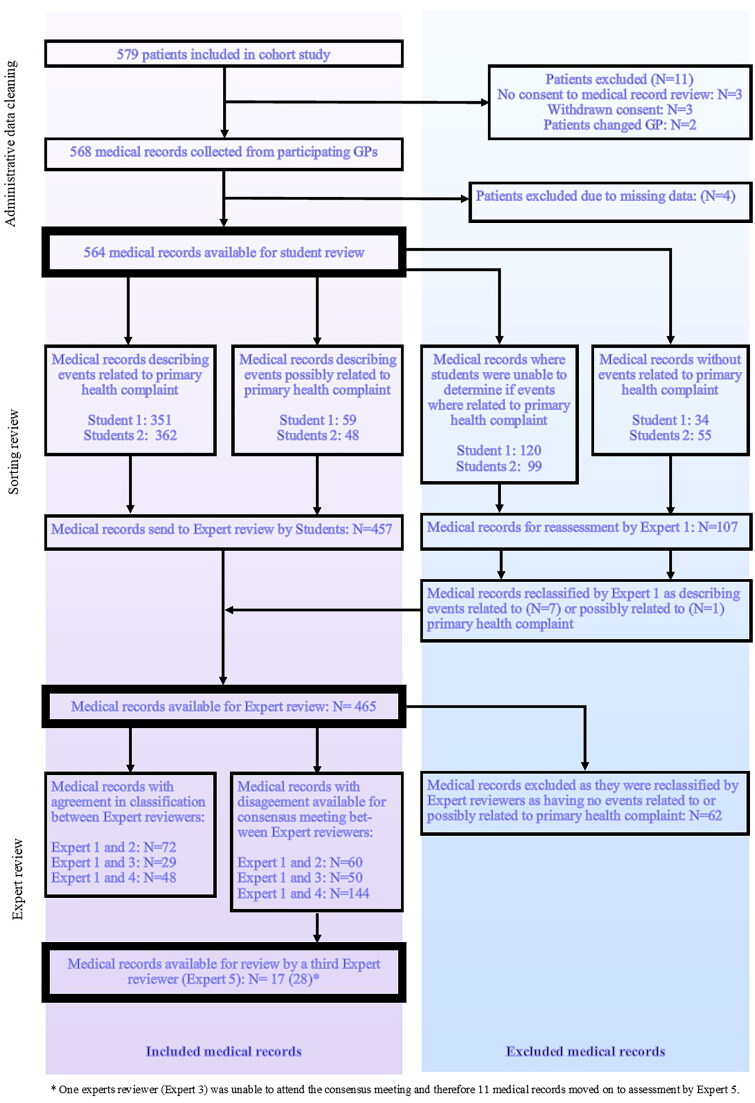
Screening of medical records.

Out of 579 patients, 15 were excluded for different reasons, and thus, 564 individual patients’ medical records were included for evaluation (Figure 2). The sorting review identified 465 medical records describing re-consultations related to or possibly the primary health complaint at the index consultation (Figure 2). This number was further reduced after review by the first expert, resulting in 403 medical records being classified as having a re-consultation relating to the initial consultation where scanning was performed.

There were classification discrepancies between the two expert reviewers regarding 254 medical records. Most of these concerned a sub-classification of the serious re-consultations (e.g. was the classification of seriousness due to the need for hospitalization or the deterioration of health itself) and were resolved through consensus between the two reviewers, resulting in 17 medical records requiring the involvement of a third expert reviewer (Figure 2).

Fourteen medical records were included for the consensus meeting: two owing to uncertainty in the classification by the third expert reviewer, one due to the classification of a serious POCUS related re-consultation, three with a possible serious POCUS-related re-consultation, three with a possible POCUS-related re-consultation, and four where the reviewers had been unable to determine the relationship between the re-consultation and the POCUS examination. In addition, the expert reviewers had asked for a specific discussion of one additional medical record to ensure correct interpretation. The results of the classifications are provided in Figure 1 and Table 1.

## POCUS-related re-consultations

In total, 11 (2.0%) POCUS-related re-consultations were identified. A narrative description of these are provided in Supplemental file 1. Six of these re-consultations were classified as serious: One was found to be causally related to a POCUS exam of the lungs at the index consultation, which led to unnecessary hospitalization and cancer suspicion, three re-consultations were found to be possible related (*N* = 2) or unlikely related (*N* = 1) to the pelvic POCUS exam at the index consultation which all led to further examinations in secondary care, and in the remaining two cases, the panel was unable to determine the relationship between POCUS at the index consultation and later surgical procedures.

In the five re-consultations that were classified as unserious, two were found to be possibly related to POCUS exams of the shoulder and gall bladder at the index consultation, one was found to be unlikely related to a POCUS exam of the shoulder, and in the remaining two events the panel was unable to determine the relationship to the initial POCUS exam of the uterus and skin, respectively. The panel assessment of appropriateness are provided in Supplemental file 1.

## Potential misdiagnosis

The expert reviewers identified possible misdiagnoses in 30 of 564 medical records, corresponding to 5.3% of all consultations where scanning was performed—most of which could be considered as false positives (Table 2). However, exact estimates of false positive diagnoses and false negative diagnoses could not be calculated due to the scarcity of the information available in the medical records. The time span between the GPs scan and subsequent imaging, along with the use of different imaging modalities, further complicated the determination of whether misdiagnosis was actually made. Nevertheless, the number of identified potential misdiagnoses was highest among the commonly performed examinations: abdominal (*N* = 8), pelvic (*N* = 8), musculoskeletal (*N* = 8), and lung (*N* = 4) (Table 2). The relative frequency of potential misdiagnoses was greatest among POCUS exams, which were rarely performed, such as bone and thyroid (Table 1).

**Table 1.** Identified potentially troublesome cases.

	Number of POCUS including specific organs *N = 822 organs scanned in 564 consultations*	Number of GPs scanning specific organs *N = 20*	Number of potentially troublesome re-consultations preceded by POCUS-exams including specific organs:			
			POCUS-related events *N (%)*	Possible misdiagnosis *N (%)*	Incidental findings *N (%)*	Potential overdiagnosis *N (%)*
Heart	35	8	0	0	0	2 (5.7)
Lung	46	10	1 (2.2)	3 (6.5)	0	1 (2.2)
Breast	3	3	0	0	0	0
Thyroid	6	1	0	1 (16.7)	0	0
Lymph nodes	4	1	0	0	0	0
Skin	34	13	0	1 (2.9)	0	0
Gallbladder	38	12	1 (2.6)	2 (5.3)	1 (2.6)	1 (2.6)
Liver	22	7	1 (4.5)	1 (4.5)	0	0
Pancreas	5	3	0	0	0	0
Abdomen (Free fluid)	16	8	0	2 (12.5)	0	0
Kidney	44	11	0	2 (4.5)	1 (2.3)	0
Urinary bladder	45	13	1 (2.2)	3 (6.7)		0
Uterus	125	17	5 (4.0)	8 (6.4)	3 (2.4)	1 (0.8)
Ovaries	51	8	4 (7.8)	3 (5.9)	1 (2.0)	1 (2.0)
Placenta	12	5	0	0	0	0
Fetus	68	17	1 (1.5)	0	1 (1.5)	0
Fluid in the cul-de-sac	6	4	0	0	0	0
Aorta	29	9	0	0	0	1 (3.4)
Veins lower limbs	16	8	0	0	0	0
Joint	98	15	3 (3.1)	6 (6.1)	0	0
Joint, puncture	17	6	0	0	0	0
Muscle	10	8	0	0	0	0
Tendon	46	14	0	1 (2.2)	0	0
Bone	8	4	0	2 (25.0)	0	0
Other*	38	12	0	3 (7.9)	0	0

Data on organs scanned originated from the registrations made by the GPs at the index consultation.

*Other included scanning of lower abdomen (*N* = 8), intestines (*N* = 6), scrotum (*N* = 5), trochanteric bursa (*N* = 5), soft tissues (*N* = 5), amnion fluid determination (*N* = 4), veins (*N* = 2), carotids (*N* = 1), ureter (*N* = 1), larynx (*N* = 1).

**Table 2.** Cases of potential misdiagnosis.

Organ scanned	POCUS finding	Subsequent finding	Comparison	Days between exams
Breast	Breast lump /Subcutaneous lipoma	Normal fibro-glandular tissue	Ultrasound performed by radiologist	8
Thyroid	Small cystic process	Normal findings	Ultrasound performed by ENT specialist	4
Abdominal ultrasound examinations				
Abdomen	Hypoechogenic structure 2x2x3 cm	Normal findings	CT scan	14
Abdomen	No ascites. Fatty enlarged pancreas with central dilated pancreatic duct	No ascites. Normal pancreas.	CT	28
Gallbladder	Small gallbladder stone with shadowing	No gallbladder stones	Ultrasound performed by radiologist	15
Kidney	Hydronephrosis/cyst 6x4x4 cm	Hydronephrosis confirmed. No cyst.	CT scan	1
Kidney	Normal findings	Large coral kidney stone	CT scan	?
Groin region	Inguinal hernias on both sides	Normal findings	CT	8
Bladder*	Pre-void bladder volume calculated to be 725 ml and postvoid 500 ml	No subsequent control		
Bladder*	Post-void bladder volume 725 ml	No subsequent control		
Pelvic ultrasound examinations				
Uterus	Retroverted uterus and no IUD visualized	Anteverted uterus and IUD *in situ*	Ultrasound performed by gynecologist	1
Uterus	No IUD visualized	IUD *in situ*	Ultrasound performed by gynecologist	21
Uterus	One large uterine fibroid with a diameter of 6 cm. Additional 2 small Uterine fibroids.	Two uterine fibroids the biggest one with a diameter of 3.6 cm. Small amount of fluid in the cul-de-sac.	Ultrasound performed by gynecologist	1
Uterus	IUD *in situ*, suspicion of uterine fibroid. Ovaries not visualized.	Uterine sequelae after cesarean section. IUD *in situ*. Ovarian cyst on the right side with a diameter of 4.8 cm.	Ultrasound performed by gynecologist	45
Uterus and ovaries	Ovarian cyst on the left side with a diameter of 2.5 cm	Two uterine fibroids the biggest one with a diameter of 2 cm. A 1 cm cyst in fundus. Ovarian cyst on the left side with a diameter of 4.4 cm.	Ultrasound performed by gynecologist	75
Ovaries	Ovarian cyst on the left side with a diameter of 5 cm. Left side 11 ovarian follicles, right side 8–10 ovarian follicles. Small amount of fluid in the cul-de-sac.	Normal findings	Ultrasound performed by gynecologist	135
Ovaries	Ovarian cyst	No cysts	Ultrasound performed by gynecologist	150
Ovaries	Ovarian cyst 25x35x35 mm	Normal findings	Ultrasound performed by gynecologist	6
Lung ultrasound examinations				
Costa and lung	Normal findings	Rib fractures and plural effusion	Ultrasound performed by emergency physician	2
Lung	Focal b-lines and bronchograms	Normal findings	Chest Xray	0
Lung	B-lines and bronchograms	Normal findings	Chest Xray	5
Lung	Focal b-lines right middle lobe side	Pulmonary congestion. Pleural effusion on both sides	Chest Xray	4
Musculo-skeletal ultrasound examinations				
Facial bones	Signs of jaw fracture	Normal findings	CT scan	0
Shoulder	Subacromial bursitis	No signs of bursitis.	Ultrasound performed by rheumatologist	40
Shoulder	Subacromial bursitis, small tears in the supraspinatus tendon, glenohumeral fluid accumulation, tenosynovitis of the biceps tendon	Complete tear of the supraspinatus tendon	Ultrasound performed by radiologist	7
Hip	Hip effusion, articular cartilage damage	Hip effusion, no articular cartilage damage	Ultrasound performed by orthopedic surgeon	42
Knee	Normal findings	Intraarticular fluid accumulation Bakers cyst	MRI	120
Knee	muscle strain	Normal findings	Ultrasound performed by orthopedic surgeon	10
knee	Normal findings	Small joint effusion	Xray	0
Plantar foot	No plantar fasciitis	Plantar fasciitis	MRI	150

Data extracted from the medical records.

* The experts suspects that the bladder volume is miscalculated due to the large volume estimate.

## Potential overdiagnosis

The expert reviewers classified four findings out of 564 clinical courses, including potential overdiagnosis corresponding to 0.7% of cases. These four cases included:

- two POCUS examinations of the heart where no subsequent imaging to confirm or dismiss the diagnosis was available, and no specific treatments were initiated in the following six months
- one examination of the lungs where the detection of pleural effusion led to hospital admission, but no further treatment and
- one of the uterus where incidental findings led to the operation.

## Incidental findings

The GPs participating in the study classified six POCUS exams as incidental findings. The expert reviewers agreed with this classification in two cases and identified three additional incidental findings through the review process. Hence, five medical records with potential incidental findings were discussed at the consensus meeting, including:

- one scanning of the uterus with the detection of a possible uterine fibroid
- one scanning of the uterus and ovaries with detection of a small ovarian cyst
- one scanning of the uterus and fetus with the detection of a multiple pregnancy
- one of the kidneys with signs of grade 1 hydronephrosis
- one of the gallbladder with a small gallbladder stone

Two of these were classified to have an unlikely net benefit (hydronephrosis and ovarian cyst), two others could not be classified in terms of net benefit (uterine fibroid and multiple pregnancy), and for the last case agreement could not be reach as to whether the gallbladder stone was in fact an incidental finding or the cause of the problem. Hence, the number of incidental findings was four, potentially five, out of 564 scans, corresponding to 0.7% or 0.9%.

## Relation of re-consultations, misdiagnosis, potential overdiagnosis, and incidental findings to scanning competence

**Figure 3.**
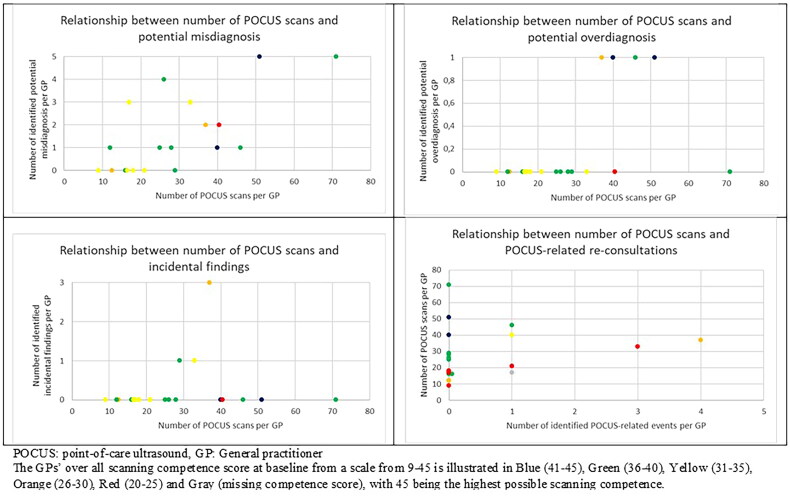
Relationship between potentially troublesome cases, number of performed POCUS scans, and scanning performance.

The number of POCUS-related re-consultations (7), potential misdiagnoses (30), potential overdiagnoses (4), and incidental findings (5) were divided among 6, 13, 4, and 3 GPs, respectively. Figure 3 depicts the relationship between the number of scans performed by each GP during the data collection month and the number of POCUS-related re-consultations, potential misdiagnosis, potential overdiagnosis, and incidental findings. The colours illustrate the level of overall scanning competence (not specifically for the given scan). Five of 11 POCUS-related re-consultations (45.5%) happened among three GPs with the lowest competence score (score < 30) who made 89 of 564 scans (15.8%). Compared to this, the ten GPs with high scores (score >35) had one POCUS-related re-consultation (9.1%) and performed 344 of the scans (61.0%).

## Relation of re-consultations, misdiagnosis, potential overdiagnosis, and incidental findings to type of scanner

Most participating GPs used mid-range laptop ultrasound devices. Only one GP used a handheld device, and one used a high-end scanner. These GPs did not stand out regarding the number of identified POCUS-related re-consultations, incidental findings, potential over- or misdiagnosis. Hence, no relationship was found between these findings and the types of devices.

## Discussion

### Statement of principal findings

This prospective 6-month follow-up study, where we reviewed medical records from 564 patients for potential inappropriate care after POCUS, describes in numbers a low risk for possible misdiagnosis (5%), potential overdiagnosis (0.7%), and incidental findings (0.7%). Still, 11 POCUS-related re-consultations were identified and described. The panel found one case of *harm* resulting from a POCUS examination of the lungs, which resulted in unnecessary hospitalization and cancer suspicion. In two other cases, possible harm followed pelvic POCUS examinations in which the GP expanded the scope of the examination to include the ovaries, resulting in incidental findings, a cascade of ensuing events, and later surgical treatment. In another case, a possible delay in diagnosis was described following the GP’s POCUS examination of the shoulder. No harms were found in the remaining seven cases, but ethical issues were raised in one case with the detection of multiple pregnancies (Supplemental file 1). Overall, potentially troublesome cases were more commonly detected among those GPs with low scanning competence at baseline.

### Strengths and limitations

Even though the participating GPs varied considerably regarding training and scanning experience, they were a selected group of early adopters [16]. The results found in this group cannot necessarily be generalized to the whole GP community. Their interest in POCUS may have resulted in a higher level of scanning competence, but they may also have been more explorative and risk-willing when using POCUS. In addition, the study was conducted in 2018 at a time when little guidance was offered for the use of POCUS in primary care. Hence, it is possible that a more restricted use of POCUS will be seen today.

As the number of detected re-consultations, potential overdiagnosis, and incidental findings were deficient, larger samples in more diverse groups of GPs are needed for statistical evaluation of these events. Limited documentation in the medical records, different imaging modalities used as the gold standard, and various lengths between the examinations made comparing the results of POCUS examinations in the index consultations and results found on subsequent imaging difficult. Hence, the 30 registered potential misdiagnoses may be an underestimation (Table 2). We found that in most cases, a six-month follow-up was appropriate, but a longer timeline is needed for some conditions, e.g. screening for abdominal aorta aneurysms.

The data used to evaluate the clinical course following POCUS were those registered by the GPs at the initial scanning and the routinely recorded medical record data six months after that. Hence, sometimes, these data need to provide more information to answer the research questions, e.g. whether an incidental finding yielded any harm or benefit for the patient.

As no existing evaluation tool was available for this study, we adapted international standards regarding adverse events from clinical studies to this context [17,18]. To avoid overlooking the effects of POCUS by merely looking at unwanted events, we started with identifying re-consultations for the same complaint in the medical records. All recurrent health problems relating to the initial consultation were identified and evaluated regarding the seriousness and causality of the initial POCUS examination. For example, if a patient initially came with knee pain and had the knee scanned and months later came again due to knee pain, this would be a re-consultation. Even if it was unrelated to the initial POCUS examination (but it would thus be registered as irrelevant). This led to a rather cumbersome registration, and the expert reviewers found the procedure time-consuming and challenging to work with. The categories were not exhaustive or exclusive, and some harms, e.g. net harm concerning incidental findings, could not be classified. In addition, it was hard to classify POCUS examinations with no medical indication. Still, the two reviewers reached consensus in most cases, and only in 17 cases did a third reviewer get involved. However, the discrepancies between reviewers’ classifications indicate the need for a better tool for evaluating patient pathways.

### Comparison to other studies

Only a few studies include quality and safety data regarding the use of POCUS in general practice [9]. Previous research has shown a high level of agreement when comparing ultrasound scans performed by GPs and experts [20]. However, high quality is not solely dependent on GPs’ ability to produce and interpret high-quality ultrasound images; their ability to use ultrasound findings appropriately in the clinical context is equally important [21].

A few early studies conducted in rural areas aimed to provide a more comprehensive evaluation. A GP clinic in rural Scotland audited quality, costs, and patients’ preferences in relation to POCUS examinations at the clinic [22]. Their overall conclusion was that the quality of the scans was adequate, the overall cost was lower when patients were scanned at the general practice clinic compared to the hospital, and patients preferred to be scanned by their GP. Likewise, a three-month follow-up of 200 POCUS examinations performed in one general practice clinic was made in Norway [23]. This study revealed an overall decrease in referrals and the necessity for blood tests. However, they also identified some diagnostic uncertainty, as doctors reported that 21 of the examinations, to some extent, yielded a false negative or false positive result, and 12 of the examinations were ill-suited for the clinical problem. Like the Norwegian study, our research was carried out at a time when little guidance on appropriate POCUS applications was available. In our study, potential misdiagnoses primarily occurred in cases where the GP suspected pathology on ultrasound, which was later ruled out, and the expert reviewers concluded that the indication for performing POCUS and its net benefit were not always clear, even though action was taken (Supplemental file 1). Some participating patients in the Norwegian study, declared that doctors put too much emphasis on medical technology, and 19% agreed that technical examinations and blood tests could interfere with the patient-GP relationship. Hence, many aspects are important to consider when evaluating the quality of POCUS in general practice.

Ultrasound is recognized as a safe medical technology [4], and our study indicated that POCUS-related adverse events in general practice were uncommon. The same conclusion was drawn from an analysis of medical malpractice in relation to POCUS use [24]. In the literature, harm is primarily associated with examinations that are overly advanced or exploratory without a predefined purpose [9]. Our results mirrored these findings: Potential overdiagnosis was found in advanced cardiac examinations, where detecting signs of disease had no impact on patient care. Incidental findings emerged when GPs strayed from the predefined purpose, such as assessing the IUD placement and scanning the sides to evaluate the ovaries. Misdiagnosis was more prevalent in advanced examinations or when ultrasound was used to investigate rare conditions. Therefore, the more problematic use of ultrasound in our study arose when the examinations departed from the concept of POCUS [25], was beyond the scope of tailored training programs [26], and beyond the curricula outlined in Delphi studies [27,28].

Still, other problematic situations may arise when GPs enter new areas. In our study, we encountered a case in which a pregnant woman had her fetus scanned for the first time by the GP when she was seven weeks into her pregnancy. There was no medical indication for performing the scan. In Denmark, pregnant women are offered prenatal screening around week 12, which includes a nuchal translucency measurement and a double test. The GP’s scan revealed a twin pregnancy, and after receiving this result, the woman decided to have an abortion. In Denmark, legal, free abortion is permitted before the end of the 12th week, making this an informed and legal decision by the woman. However, the GP’s opportunistic screening provided the woman with information about the twin pregnancy before she had undergone prenatal screening. Had the twin pregnancy been identified during prenatal screening, she might not have chosen an abortion, as the obstetric consultation could have presented her with other options, such as fetus reduction or social support. Thus, opportunistic scanning in early pregnancies may lead to new ethical challenges in general practice.

### Meaning of the study

This study demonstrated that POCUS scanning performed by GPs was generally safe; however, there was a slight risk of unnecessary subsequent examinations or potential harm in a few cases as potential misdiagnosis (30 cases) primarily caused by false-positive diagnoses, incidental findings (5 cases), and potential overdiagnosis (4 cases), led to follow-up imaging in secondary care. Certain areas, such as pelvic scans that included the ovaries, may be particularly susceptible to misdiagnosis. The findings should be interpreted cautiously due to the small GP population, low incidence of events, and, for some conditions, an insufficient follow-up time. Still, the study provides insights into the early stages of implementation and the behaviour of early adaptors of ultrasound technology, which may be used to inform the development of strategies for the next stages of implementation.

### Unanswered questions and future research

This external medical audit was conducted without explicit quality indicators, as no best-practice clinical guidelines were available [13]. The evaluation was conducted anonymously, so participating GPs did not receive feedback. The GPs were expected to navigate according to the primum non nocere (Latin for “first do no harm”) and ALARA (as-low-as-reasonably-achievable) principles. Still, unforeseen harms and redundant examinations occurred, calling for more explicit recommendations [29] and evaluation tools with built-in feedback to be developed [30]. During our review process, instances of opportunistic screening, such as for a fetus or the aorta, were identified but not necessarily classified. The reviewers found such cases worrisome as they could result in low-value and harmful care and exacerbate the inverse care law [31]. Therefore, future studies should include a “no medical indication” category to assess whether low-value care or harmful care arises from implementing POCUS in general practice where there is no medical indication. Potential issues related to scanning safety should be further explored in a larger patient population and a more diverse group of GPs. Near-miss situations should also be documented to enhance the ability to prevent harm.

## Supplementary Material

Supplemental file 1 Narrative description of POCUSrelated reconsultations.docx

## Data Availability

The data used in this study was medical records containing highly sensitive information about participating patients. Therefore, the dataset is not made available. Data extraction sheets can however be made available upon reasonable request.
