# Supplementary material for: Does point-of-care ultrasound examination by the general practitioner lead to inappropriate care? A follow-up study
Source: Scand J Prim Health Care. 2025 Apr 10;43(3):613–25. doi: 10.1080/02813432.2025.2487095 (PMC12377149; doi:10.1080/02813432.2025.2487095)
Supplement: Supplemental file 1 Narrative description of POCUSrelated reconsultations.docx [file IPRI_A_2487095_SM9488.docx]

**Supplemental file 1: Narrative description of POCUS-related re-consultations**

| Classification of event | Relation to POCUS classified as | Description of patient pathway | Comments and classification following the consensus meeting |
| --- | --- | --- | --- |
| Possibly POCUS related re-consultation | Possibly related | The patient had the liver and gall-bladder POCUS examined at a follow-up consultation due to elevated liver-enzymes. The GP recorded that the gallbladder had a regular smooth surface, and the liver was without visual processes. There was no fluid in the hepatorenal fossa. The GP registered that POCUS led to a change in diagnosis, but not in patient management. The GP also register an uncertainty in the findings.  The patient was referred on to the radiology department on the suspicion of liver pathology. The radiologists’ ultrasound examination revealed a 3 mm gallbladder polyp, which lead to the patient being enrolled in a follow-up program with repeated ultrasounds examinations. No explanation for the elevated liver enzymes was found, but possible explanations could be previous prostate cancer, treatment with statins, and a high alcohol consumption. | The panel are unsure whether the patient would have been referred on to an ultrasound examination if the GP had not used POCUS.  The patient was enrolled in a follow-up control program, despite a benign finding below the 5 mm cut-off for gallbladder polyps mentioned in guidelines.  The Gallbladder polyp was not seen on the GPs POCUS exam.  No harm followed the GPs management of the patient, but the POCUS seemed to be redundant in the diagnostic process. |
| Possibly POCUS related re-consultation | Possibly related | A patient consulted the GP because of shoulder pain. The physical examination pointed to subacromial impingement. The GP examined the shoulder with POCUS and found fluid accumulation in the subacromial bursa and an irregular supraspinatus tendon with impeded movement under the acromion. The GP registered that POCUS led to a change in diagnosis and management and a certainty in findings. The patient was referred on to an orthopedic surgeon on the suspicion of cuff-rupture.  Waiting to see the surgeon, the patient consults the GP again and received a steroid injection. Later the orthopedic surgeon found signs of arthropathy and arthrosis resulting in planned surgery to insert a prosthesis. | It is uncertain whether it was the POCUS performed by the GP that led to the referral and subsequent operation.  It is not known if an operation will lead to improved or impaired health outcome of the patient.  No harm followed the GPs management of the patient, POCUS was used sensible in the diagnostic process to qualify the referral. |
| Possibly POCUS related re-consultation | Unlikely related | A patient consulted the GP because of shoulder pain. During the physical examination the patient had difficulties with internal rotation in the shoulder joint. Hawkin’s and Neer’s tests were positive and there was a positive isometric test of supraspinatus. The external rotation in the shoulder joint was not described. The GP examining the shoulder using POCUS found a thickened subacromial bursa. The GPs subsequently registered a change in diagnosis and management of the patient and a certainty in findings. The patient was referred to a physiotherapist for treatment for shoulder impingement. The physiotherapist saw the patient 2-3 weeks later and based on the physical examination of the patient, the physiotherapist suspected capsulitis and asks the patient to re-consult the GP. The GP then referred the patient to a rheumatologist, who two weeks later found a normal ultrasound examination, but a clinical suspicion of capsulitis. Therefore, the patient received a steroid injection. MRI later confirmed capsulitis and fluid accumulation in the subacromial bursa. | We are not able to determine whether there were other signs of capsulitis at the index consultation.  However, the POCUS finding of a thickened subacromial bursa may have resulted in a delay in the capsulitis diagnosis for this patient.  Hence, harms (in terms of delayed diagnosis) caused by the use of POCUS in this case can not be ruled out. |
| Serious POCUS related re-consultation | Causal related | A patient was seen in general practice due to pneumonia not sufficiently responding to antibiotic treatment. GP performed POCUS of the lungs revealed a pleural fluid accumulation posterior inferior on the left side. The patient was admitted to the hospital on suspicion of empyema. The GP registered that POCUS changed the patient’s diagnosis and the management plan. The GP also register a certainty in findings.  At the hospital, they performed a chest x-ray, in which pleura effusion was not seen and the patient was discharged. Four days later the patient visited the GP where POCUS again showed pleural effusion on the left side. The GPs consulted a lung-specialist, who recommended a fast-track CT-scan on the suspicion of lung cancer. The CT-scan shows no sign of malignancy, but sequelae after a lung infection and pleural effusion on the left side.  The GP and the hospital continued to follow-up on the patient condition in the following weeks where the C-reactive protein level continued to drop. | The panel agrees that the detection of pleural effusion made by the POCUS examination performed by the GP at the index consultation was the cause of an unnecessary admission to hospital and later the fast-track referral for cancer.  However, the pleural effusion was there, and it is plausible that the patient would have been referred on from the GPs office if POCUS had not been available.  POCUS is more sensitive than x-ray for detecting pleural effusion. Still. the detection of the fluid is this patient had no impact on the treatment instead it led to extra examinations and hospital admission. |
| Serious possibly POCUS related re-consultation | Possibly related | A patient had a check of her IUD three months after IUD insertion at her GP. Heavy bleeding and pain during menstruation had stopped after the insertion of the IUD, but the patient experienced spotting. Using POCUS the GP found the IUD in place, no uterine fibroids, and an adnexal cyst on one side. The GP registered that the POCUS led to a change in the diagnosis and plan for the patient, but also uncertainty in the findings. A follow-up POCUS was arranged three months later, where the GP found a hemorrhagic cyst on the other side (length 35 mm) . As the patient had had a hemorrhagic cyst before, she was referred to a gynecologist.  Six days later, the patient was seen by the gynecologist. The patient thought that she was referred on the suspicion of cancer, was scared, nervous, and she described having pain in her entire lower body. The gynecologist found no suspicion of cysts, but instead suspected that the IUD was misplaced in the cervix and therefore performed a saline infusion sonography of the uterus. This examination did not reveal the placement of the IUD and the patient was referred to hospital.  At the hospital, sonography showed a correctly located IUD. Due to heavy bleeding described by the patient an endometrial resection was performed.  The patient was subsequently followed by the GP and continued to have spotting. | Ultrasound was here used to ensure correct placement of an IUD following insertion in the uterus.  This GPs expanded the ultrasound examination to the sides beyond the uterus and found an incidental adnexal cyst. This incidental finding did not result in referral at the index consultation, but led to a follow-up consultation. At the follow-up the patient’s spotting symptoms persisted.  It is plausible that the patient would have been referred regardless of the POCUS exam due to the persisting symptoms.  The panel cannot conclude whether the referral was a result of the POCUS exam performed by the GP. However, misdiagnosis does occur possibly at the first POCUS, and definitely at the second POCUS. |
| Serious possibly POCUS related re-consultation | Possibly related | A patient consulted the GP with new bearing-down feeling and heavy bleeding. The GP found an enlarged and irregular uterus. The GP used POCUS, but had difficulties visualizing the uterus and wrote that this could be due to fibroids. The ovaries were described as normal, and no fluid accumulation was seen in the cul-de-sac. The GP registered that POCUS led to no change in diagnosis or management of the patient and uncertainty in the sonographic findings. The GP took a cervix smear test and referred the patient to a gynecologist. Blood samples including hemoglobin and a CA125 test were normal.  The patient had to wait four weeks to see the gynecologist, and one week after the index consultation, the GP re-examined the patients with POCUS in an attempt to visualize the structures better. The GP now suspected an ovarian cyst (length 35 mm) and the patient was fast-track referred to hospital on the suspicion of cancer.  At the gynecology department they found a normal pelvic examination and on ultrasound they described a small intramural fibroid. They interpreted the patient symptoms as perimenopausal bleeding disturbances and recommend an IUD if needed. No further action was taken. | The first POCUS performed by the GP turns out to be difficult and inconclusive, but it did not entail harms as the patient was referred to a gynecologist for evaluation.  The second POCUS did however result in a fast-track cancer suspicion based on misdiagnosis.  The panel points out that the second POCUS exam may be a result of the first POCUS exam and therefore the harms are possibly related to the POCUS performed at the index consultation. |
| Serious possibly POCUS related re-consultation | Unlikely related | A patient consulted her GP with continuous daily bleeding two months after insertion of an IUD. The pelvic examination was normal. POCUS examination showed the IUD to be in place, no uterine fibroids, or adnexal cysts, but the GP spotted a small hyperechoic structure measuring 25 mm and suspected it to be a small uterine fibroid. The GPs registered that the POCUS entailed a shift in the diagnosis and management for the patient, but an uncertainty in the sonographic findings and the patient was referred to a gynecologist.  One month later, the patient was seen by the gynecologist, who also found a hyperechoic structure now measuring 40 mm. Blood samples including CA125 were normal. Two months later the structure has mixed echogenicity and measured 5 x 3.5 cm. The patient was referred to the hospital for operation. Another two months later (five months after the index consultation) the patient was seen at the hospital, here the structure measured 40 mm and they suspect it to be a dermoid cyst. One month later a new ultrasound examination raised suspicion of the structure being a pedunculated fibroid and operation was performed. The pathological result was not available in the medical record, but the GP later noted in the medical record that it was a fibroid. | The GPs performed POCUS to control the position of the IUD, but expanded the examination to look to the sides and an incidental finding was detected. The GPs was unsure regarding the interpretation of the findings and referred the patient on – and a long diagnostic pathway followed.  The hospitalization and operation were not a direct result of the initial POCUS examination rather a cascade of events.  As there is no certain histological elaboration, it cannot be determined whether harms followed the GPs POCUS findings, and the net benefit of the incidental finding cannot be determined. However, during the months of follow-up there was no notes in the medical records suggesting that there had been any malignancy.  The panel concluded that in all likelihood this is a case of over-detection and over-treatment. |
| Serious re-consultation | Unable to determine | A pregnant patient consulted her GP with another health-related concern. As part of the consultation, the GP performed a transvaginal POCUS and found that the patient was expecting twins. The GP registered that POCUS entailed a change in diagnosis, but not in management of the patient. The forthcoming standard prophylactic pregnancy consultations was scheduled and the possibility or a section in GA 38+0 was discussed with the patient.  The patient was overwhelmed with the news of a gemelli pregnancy and the next day she re-consulted the GP with a decision to have an abortion. There are several consultations the following weeks and in the end the patient chose to have an abortion. Five months later she became pregnant with a singleton pregnancy, which she kept. | Assessing the number of fetuses and heartbeat may lead to unexpected findings and ethical dilemmas.  In this case POCUS revealed a twin pregnancy and the woman chose to have an abortion. We do not know the circumstance regarding the family, etc. and cannot judge this choice.It was an informed choice, and the patient was given support and re-consultations in the weeks following the index consultation.  We do not know whether the patient would have chosen to keep the pregnancy if the information was given at a later stage in the pregnancy (within the free abortion time frame which was 12 weeks gestation in Denmark at the time).  Hence, the panel is unable to determine whether the hospitalization and the abortion was a direct result of the POCUS performed by the GP. |
| Serious re-consultation | Unable to determine | The Patient was seen in general practice and the GP performed a POCUS exam of the bladder, which was registered as having led to a change in management. Later the patient has an operation for prostate hypertrophy. | There is no note in the medical record from the index consultation, so the panel cannot evaluate the clinical course. |
| Re-consultation | Unable to determine | A patient consults her GP because of a bearing-down feeling in the lower abdomen. The patient is known to have a uterine fibroid. In the pelvic examination the GPs found an enlarged uterus and on POCUS the fibroid was 6 cm compared to 4 cm at the last examination. The GP also found two smaller fibroids and the patient was referred to a gynecologist.  The GP register a change in diagnosis and patient management following POCUS and a certainty in sonographic findings.  The patient was seen by the gynecologist the next day, The gynecologist founds two fibroids the largest one measuring 3.6 cm andfluid accumulation in the uterus. One month later, a new ultrasound examination was conducted where several fibroids were found the largest measuring 5 cm and the fluid inside uterus was still there. A biopsy of the endometrium was made, and the patient was scheduled for a repeated ultrasound two months later. After additional two months, the ultrasound findings were the same and no further controls were scheduled. | The panel is unable to determine if it was the POCUS examinations or the finding in the pelvic examination during the index consultation that gave rise to the referral for the gynecologist.  There are no harms in this patient’s clinical course, but the panel noted that the redundant examinations may be a waste of resources. |
| Re-consultation | Unable to determine | A patient consults a GP because another doctor had noticed a swelling above the patients’ right clavicula. In the physical examination the GP found a firm swelling in connection with the right sterno-clavicular joint. The sonographic findings of the POCUS examination performed by the GP was not described in the medical record. However, the GP registered that POCUS entailed a change in diagnosis, but not in management and an uncertainty in the findings. The GP refers the patient to the radiology department. Five weeks later a CT-scan was performed which raises the suspicion of an apical lung tumor. Several scans follow and a bronchoscopy, but malignity was not found or confirmed. | There is no description of the POCUS finding in the index consultation, so the panel was unable to determine whether the referral for the radiology department was a result of the scan or the physical examination. However, the panel found it plausible that the patient would have been referred regardless as the suspicion had been raised by a colleague prior to the index consultation. |
